# The Post‐Cholecystectomy Gut Microbiota Follows a Time‐Varying Change—A Pilot Study

**DOI:** 10.1002/jgh3.70329

**Published:** 2025-12-28

**Authors:** Yu‐Tse Chiu, Fu‐Jen Lee, Chen‐Ya Kuo, Po‐Chih Yang, Jia‐Uei Wong, Kai‐Shun Liang, Ching‐Hung Tseng, Jaw‐Town Lin, Chun‐Ying Wu, Chi‐Yang Chang

**Affiliations:** ^1^ Division of Gastroenterology and Hepatology, Department of Internal Medicine Fu Jen Catholic University Hospital New Taipei City Taiwan; ^2^ Institute of Biomedical Informatics, National Yang Ming Chiao Tung University Taipei Taiwan; ^3^ School of Medicine, College of Medicine, Fu Jen Catholic University New Taipei City Taiwan; ^4^ Division of General Surgery, Department of Surgery, Fu‐Jen Catholic University Hospital New Taipei City Taiwan; ^5^ Graduate Institute of Clinical Medicine, College of Medicine, National Taiwan University Taipei Taiwan; ^6^ Germark Biotechnology, Co., Ltd. Taichung Taiwan; ^7^ Division of Gastroenterology and Hepatology, E‐Da Hospital Kaohsiung Taiwan; ^8^ Division of Translational Research, Taipei Veterans General Hospital Taipei Taiwan

**Keywords:** 16S rRNA sequencing, colorectal cancer, gallstone, gut microbiota, post‐cholecystectomy diarrhea

## Abstract

**Background:**

Gut microbiota is proposed to be a key factor for post‐cholecystectomy diarrhea (PCD). Long‐term follow‐up showed decreasing PCD over time, suggesting microbiota change is time‐dependent. Our study aimed to analyze gut microbiota and PCD at different times to clarify their time‐varying features.

**Methods:**

Patients aged 20–70 receiving laparoscopic cholecystectomy were recruited prospectively. Subjects with prior abdominal surgery, major systemic diseases, use of antibiotics, probiotics, or proton pump inhibitors within 1 month were excluded. Stool was analyzed at baseline (BL) and 6th, 12th month post‐surgery. Microbial diversity was assessed with R phyloseq package, and differential abundance of taxonomic composition with LEfSe/metagenomeSeq.

**Results:**

Ten patients were enrolled. Bristol stool scale peaked at 1 M (3.9 → 4.5 → 3.4; *p* = 0.167/0.059 for BL/1 M and 1 M/12 M). Diversity was significantly lower at 6 M in observed and Chao1 (*p* = 0.036/0.002). LEfSe indicated bimodal changes in *Lactobacillus*/*Ruminococcus*, with consistently increasing *Fusobacterium*. MetagenomeSeq revealed higher *Prevotella* at 6 M. These taxa changes have been implicated in prior studies on PCD and increased colorectal cancer after cholecystectomy. At phylum level, the decreased *Firmicutes*/*Bacteroidetes* at 6 M suggests different mechanisms than irritable bowel syndrome.

**Conclusions:**

This study showed time‐varying trends of microbial diversity and composition. Larger studies are needed for further validation.

## 1 Introduction

The gallstone disease remains a major public health issue despite the advances in modern medicine. Globally, 6% of the population have gallstone diseases, causing substantial health and economic burden [1]. Laparoscopic cholecystectomy is the treatment of choice for symptomatic gallstone diseases. Nevertheless, up to 35.6% of patients experienced post‐cholecystectomy diarrhea (PCD) [2], greatly impacting the post‐surgery quality of life. Featured by urgency, increased frequency and water content of stool, the etiology of PCD is likely to be multifactorial. Previous studies had demonstrated elevated bile acids in stool among patients with PCD, highlighting the possible mechanism of bile acid malabsorption [3]. Recent research also found the associations between bowel habitus change after cholecystectomy and the alteration of gut microbiota, implying the role microbiota played in the development of PCD [4, 5, 6, 7]. On the other hand, current evidence shows that the prevalence of PCD may change over time. The proportion of PCD declined from 25.2% 1 week after cholecystectomy to 5.7% 3 months later in one prospective cohort [8]. Another prospective cohort showed that compared with pre‐cholecystectomy status, the most evident change of bowel habitus 2–6 months after cholecystectomy is the perception of less constipation, with only a modest increase of bowel movement from seven times per week to eight times [9]. In correspondence with these phenomena, the time‐varying composition of primary/secondary bile acids was observed. Even though bile acid composition changed significantly shortly after cholecystectomy [4], long‐term follow‐ups of 5–8 years showed the total bile acid pool size and the composition remained similar through various pathways, such as the enhanced turnover rate of deoxycholic acid (DCA) which counterbalances the accelerated conversion of cholic acid to DCA [10]. Above literature indicated that PCD itself might be transient in most post‐cholecystectomy populations. However, there are no existing data regarding the time‐dependent feature of gut microbiota after cholecystectomy. All the previous investigations focused on the cross‐sectional comparison between the post‐cholecystectomy populations and the healthy control [4, 5, 6, 7]. Therefore, in this study, we aimed to perform a longitudinal analysis of gut microbiota and related clinical parameters, including changes in bowel habits, among post‐cholecystectomy populations at different time points.

Another important issue after cholecystectomy is the incidence of colorectal cancer (CRC). The increased risk of right‐sided colon cancer was first proposed in the 1980s, with a relative risk of 1.87 compared with the left‐sided one [11]. The risk declines with increasing distance from the opening of the common bile duct, implying that the change of intestinal exposure to bile might play a role [12]. Subsequent research yielded controversial results [13, 14], and some postulated that the alteration in gut microbiota after cholecystectomy and its interaction with bile acid metabolism are the main factors [15, 16]. Therefore, the change of microbiota that had been reported to be associated with the development of CRC in literature was also be investigated [17, 18, 19].

## 2 Methods

### 2.1 Subjects

Patients aged between 20 to 70 year‐old who presented with gallstone‐related symptoms and planned to receive laparoscopic cholecystectomy were recruited since July 2019. Subjects with a medical history of intra‐abdominal operation, major liver/kidneys/heart diseases, or use of antibiotics, probiotics, or proton pump inhibitors (PPI) within 1 month were excluded. Informed consents were obtained from all participants. As a pilot study, a goal of total 10 enrollments from the Fu Jen Catholic University Hospital (New Taipei, Taiwan) was determined. Demographic data including age, gender, and body mass index (BMI) were recorded. The Bristol stool form scale (BSS) and defecation frequency were documented in the different time period for correlation with the microbiota change.

### 2.2 Sample Collection

Stool was sampled within 1 month before laparoscopic cholecystectomy (as the baseline) and 1st/3rd/6th/12th month after operation (referred to as BL/1 M/3 M/6 M/12 M in the following); the stool samples of BL, 6 M, and 12 M were analyzed in this pilot study. The stool collection kit included a collection box designed for use with a seated toilet, along with a coolant pack, insulated bag, and courier label. After sample collection, participants could directly send the sample to the testing facility via courier. Detailed instructions were provided to participants in an informational leaflet. Bacterial DNA was extracted using QIAamp Fast DNA Stool Mini Kit (Qiagen, MD, USA). The DNA yielded from samples was directly used in polymerase chain reaction (PCR) assays and sequencing library construction, of which the amount and quality were determined with NanoDrop ND‐1000 (Thermo Scientific, Wilmington, DE, USA). Extracted DNA was stored at −80°C prior to 16S rRNA sequencing. Bioinformatics analysis of the stool samples was described in Supporting Information: Digital Content 1 in detail.

### 2.3 Outcomes

The main outcome focused on the change in microbiota at BL, 6 M, and 12 M. Different aspects including bacterial diversity and the change of taxonomic composition were analyzed. The genera that had been reported to be associated with PCD or CRC will be specifically discussed.

### 2.4 Statistical Analysis

Continuous variables are expressed as mean with standard deviation (SD) and analyzed by Wilcoxon rank sum test, whereas categorical variables expressed with proportion and examined by Chi‐squared test. The Wilcoxon Signed‐Rank Test was used for comparison of paired samples at different time points, such as Bristol stool scale and defecation frequency. The above statistical analyses were conducted using StataSE 14 or python through Google Colab. Diversity indices including Observed, Chao1, Shannon, Simpson, and InvSimpson were estimated using the *phyloseq* package in *R software*, which facilitated data processing, visualization, and diversity analysis [20], and Kruskal‐Wallis Rank Sum Test was used to see if significant difference exists across different time points. Linear discriminant analysis (LDA) effect size algorithm (LEfSe), which consists of Kruskal‐Wallis test to detect features with significant differential abundance, Wilcoxon signed‐rank test to determine biological consistency, and LDA to estimate the effect size of each differentially abundant feature, was used to identify bacteria with differential abundance between each time point. MetagenomeSeq was used for the same purpose, applying cumulative sum scaling (CSS) normalization to control for measurement bias across taxonomic features. It also uses a zero‐inflated Gaussian model to address biases in differential abundance testing caused by the under‐sampling of microbial communities [21]. Any additional statistical methods used will be described in the following text. All tests were two‐sided, and a *p* value less than 0.05 was deemed as statistically significant.

### 2.5 Ethical Consideration

Our study was approved by the institutional ethics committee of Fu Jen Catholic University Hospital (FJUH‐IRB number: FJUH109068). This study was performed in accordance with the Declaration of Helsinki. This work was supported by grants from Fu Jen Catholic University and Liu‐Kung Agriculture Foundation (project number: 7100086). There is no other financial support or conflict of interest to declare.

## 3 Results

**FIGURE 1:**
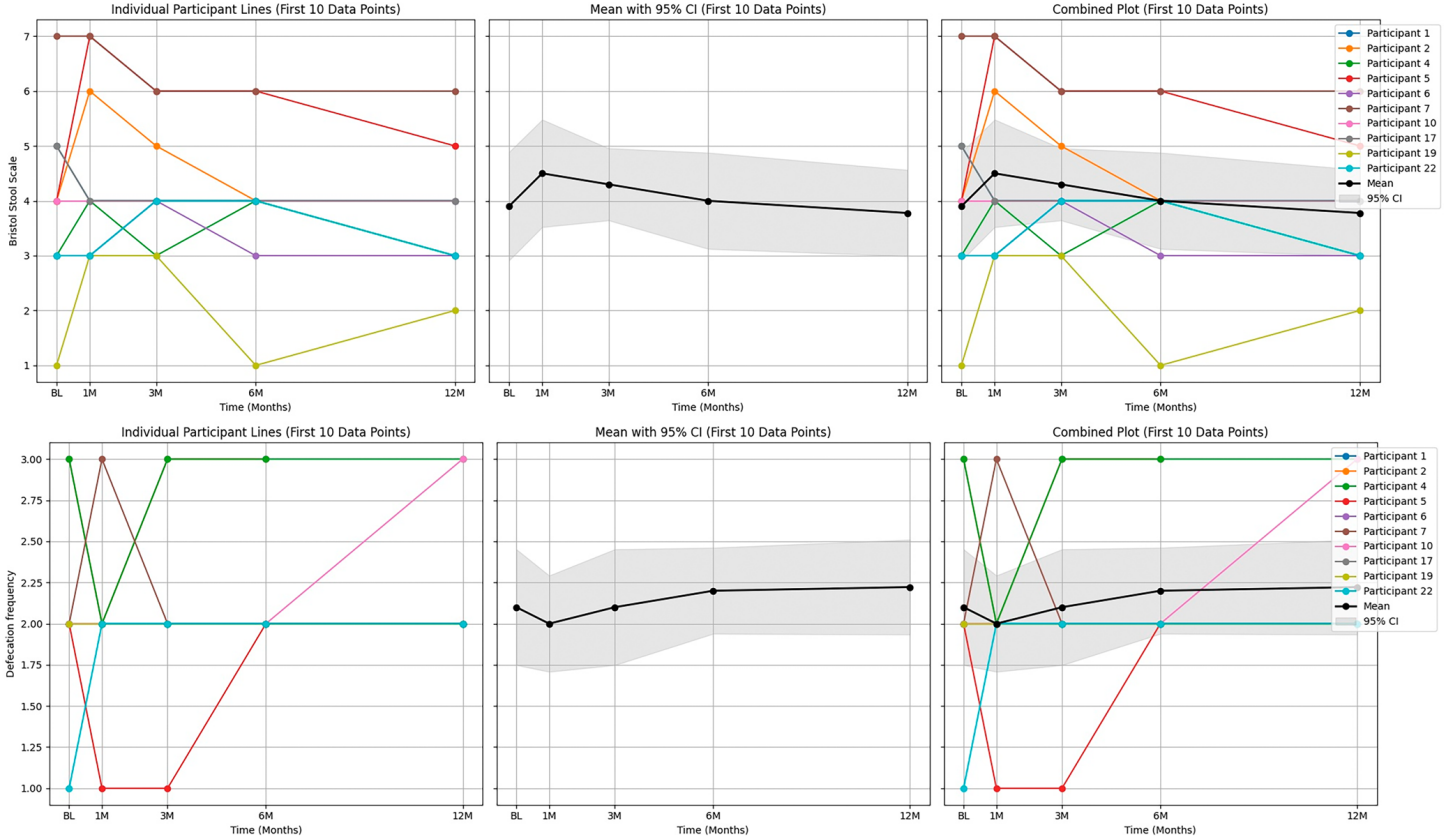
The Bristol stool scale and defecation frequency at each time point.

From July 2019 to January 2020, a total of 22 patients were screened. Twelve of them were excluded from this study, one for intra‐abdominal operation, one for use of PPI within 1 month, three who didn't receive cholecystectomy later, five for failure of stool sampling before the operation, and two for withdrawal of informed consent owing to personal reasons. At last, total 10 patients were recruited. The stool sample collection was completed in December 2020. The basic characteristics of the participants are listed in Table 1. The BSS and defecation frequency at each time point were shown as Figure 1. There was substantial variability in the defecation frequency across the participants, while the BSS demonstrated a distinct trend over time. The BSS reached peak at 1 M from baseline and then declined gradually to the lowest point at 12 M (3.9 → 4.5 → 3.4; *p* = 0.167 between BL and 1 M and *p* = 0.059 between 1 M and 12 M).

**TABLE 1:** Basic characteristics.

Age (years), mean ± SD (min–max)	46.7 ± 10.2 (37–67)
Male, *n* (%)	5 (50%)
Body weight (kg), mean ± SD (min–max)^a^	72.6 ± 18.4 (48–108)
Body height (m), mean ± SD (min–max)^a^	1.63 ± 9.54 (1.50–1.80)
BMI (kg/m^2^), mean ± SD (min–max)^a^	27.0 ± 4.40 (21.3–35.7)
Medical history	
Hypertension, *n* (%)	2 (20%)
Diabetes, *n* (%)	1 (10%)
Dyslipidemia, *n* (%)	3 (30%)
Alcohol History	
No more than once per week (none or social)	10 (100%)
Smoking	
Never	7 (70%)
Current smoker	2 (20%)
Quitter	1 (10%)

^a^ One patient's profile of weight, height, and BMI was missing.

**FIGURE 2:**
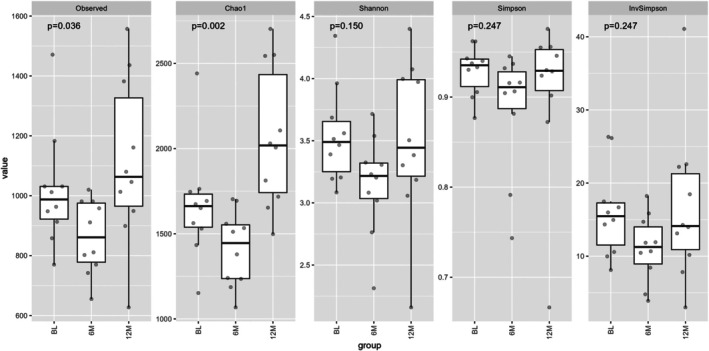
Alpha diversity at BL, 6 and 12 M.

**FIGURE 3:**
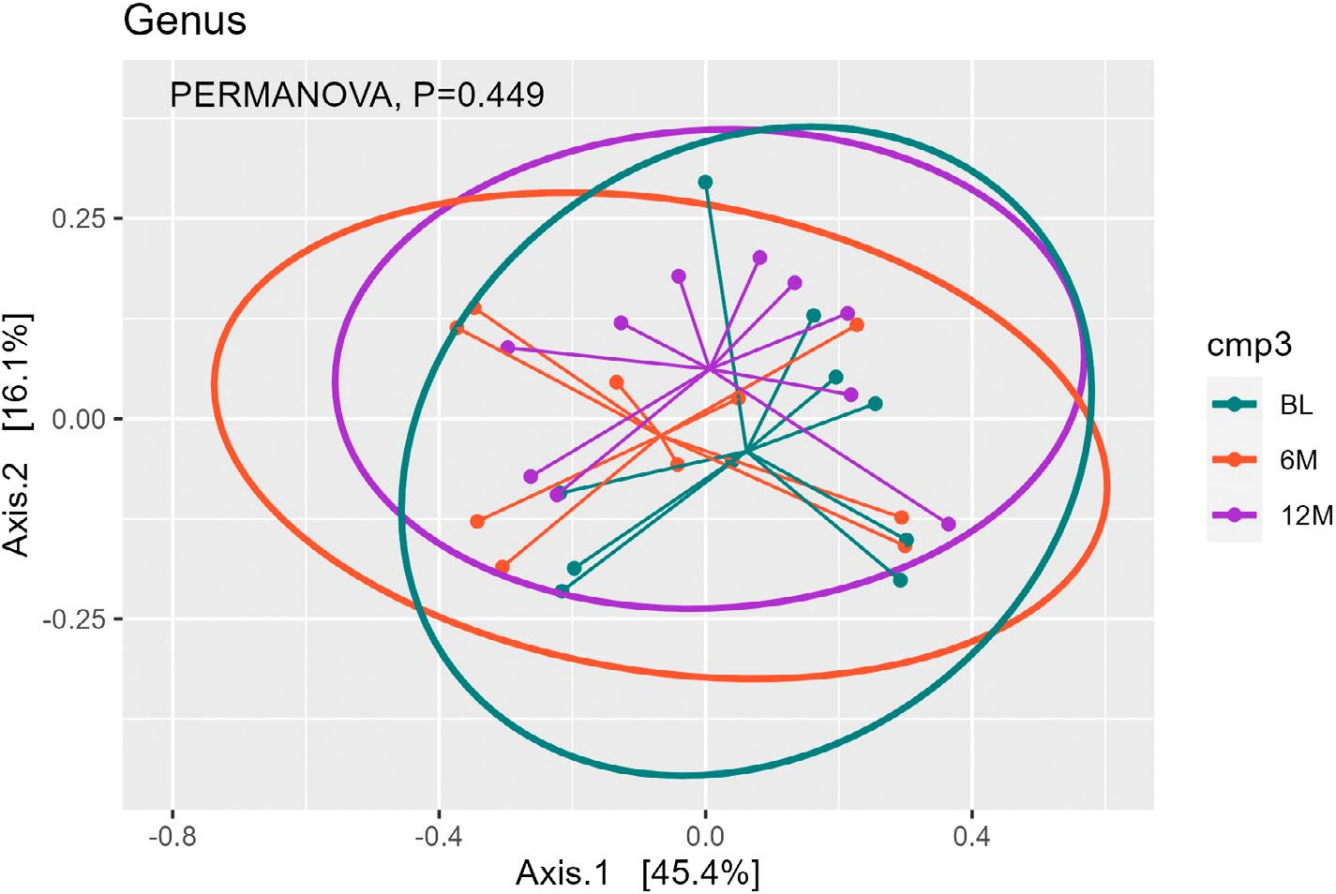
Bacterial composition in terms of principal coordinate analysis (PCoA).

Alpha diversity quantified the species in terms of operational taxonomic units (OTUs) per sample based on microbiota composition (Figure 2). Observed and Chao1 showed species richness, and the diversity at 6 M was significantly lower in both (*p* = 0.036 and 0.002, respectively). On the other hand, Shannon, Simpson, and InvSimpson considered both richness and evenness, which all showed no significance, although a similar trend was observed among them. Differences in bacterial taxa between each time point were analyzed using principal coordinate analysis (PCoA) on the genus level by Bray‐Curtis distance measure of all samples based on OTU‐level relative abundance profiles using the R package ade4 (Figure 3) [22], and statistics were performed using PERMANOVA. Although the taxonomic profiles of BL and 12 M seemed more similar to each other, there was no significant difference between them and 6 M (*p* = 0.245 for BL vs. 6 M; *p* = 0.394 for 6 M vs. 12 M).

**FIGURE 4:**
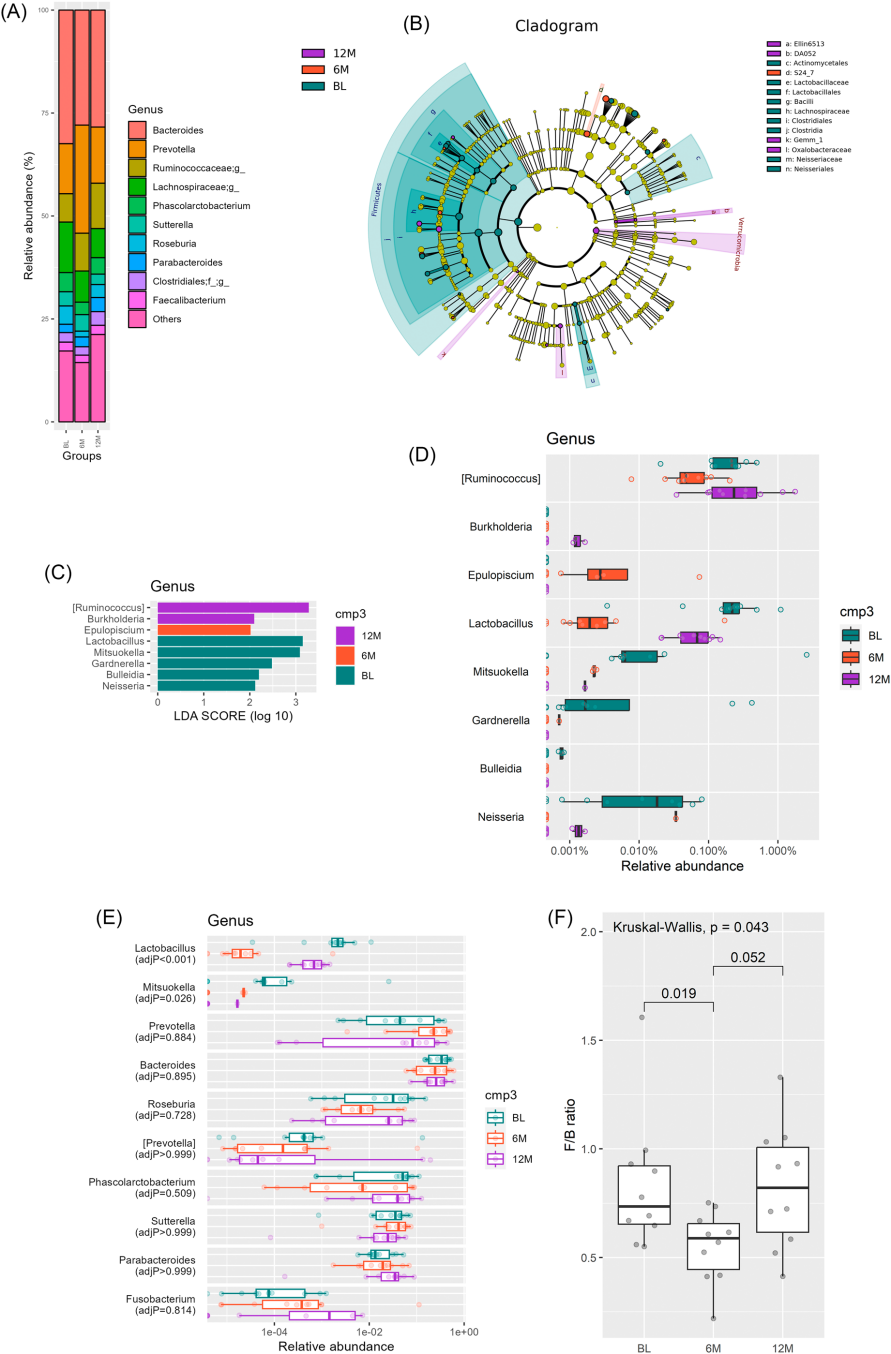
The microbiota abundance at different time points. (A) Bacterial taxa at different time points at genus level. Genera with top‐10 average abundance were included, with the rest collapsed as others. (B) Cladogram depicting taxa with significantly higher abundance at specific time points (BL, 6 M, 12 M), as determined by LEfSe analysis. Taxa that are more abundant at 12 months (12 M) are highlighted in purple, at 6 months (6 M) in red, and at baseline (BL) in green. (C) The 8 genera with significantly higher abundance at specific time points. (D) This boxplot showed the relative abundance of the 8 genera with significance at LEfSe. (E) The metagenomeSeq summarized ten genera with maxnonzeroprop > 0.5, meandiff > 0.001; the two with adj *p* values < 0.05 were prioritized; others were displayed in order of meandiff. (F) The F/B ratio showed an interval change, with a significant decline at 6 M compared with BL and 12 M.

The microbiota abundance, from phyla to genera, was different between each time point. Figure 4A showed profiling of bacterial taxa on genus level at different time points. LEfSe analysis was applied with *α* = 0.05 (Kruskal–Wallis and Wilcoxon tests) and effect size threshold of 2 on LDA to compare the relative abundance of each taxon between groups. The results were visualized using a cladogram (Figure 4B). This analysis identified 8 genera with significantly higher relative abundance at specific time points (Figure 4C and Table S1), and Figure 4D showed relative abundance of them at each time point. Then, we implemented metagenomeSeq, and those who met the following conditions would be given priority for presentation: maximum proportion of non‐zero relative abundances (maxnonzeroprop) > 0.5, average of the absolute differences in relative abundance (meandiff) > 0.001, and adjusted *p* values in the Benjamini–Hochberg procedure (adj *p* values) < 0.05. Based on the ranking of the meandiff, the top ten genera were listed as Figure 4E and Table S2. Among them, only *Lactobacillus* and *Mituokella* exhibited an adjusted *p* value < 0.05. At the phylum level, the *Firmicutes*/*Bacteroides* ratio (F/B ratio) demonstrated an interval change, with a significant decline at 6 M compared with BL and 12 M (Figure 4F).

**FIGURE 5:**
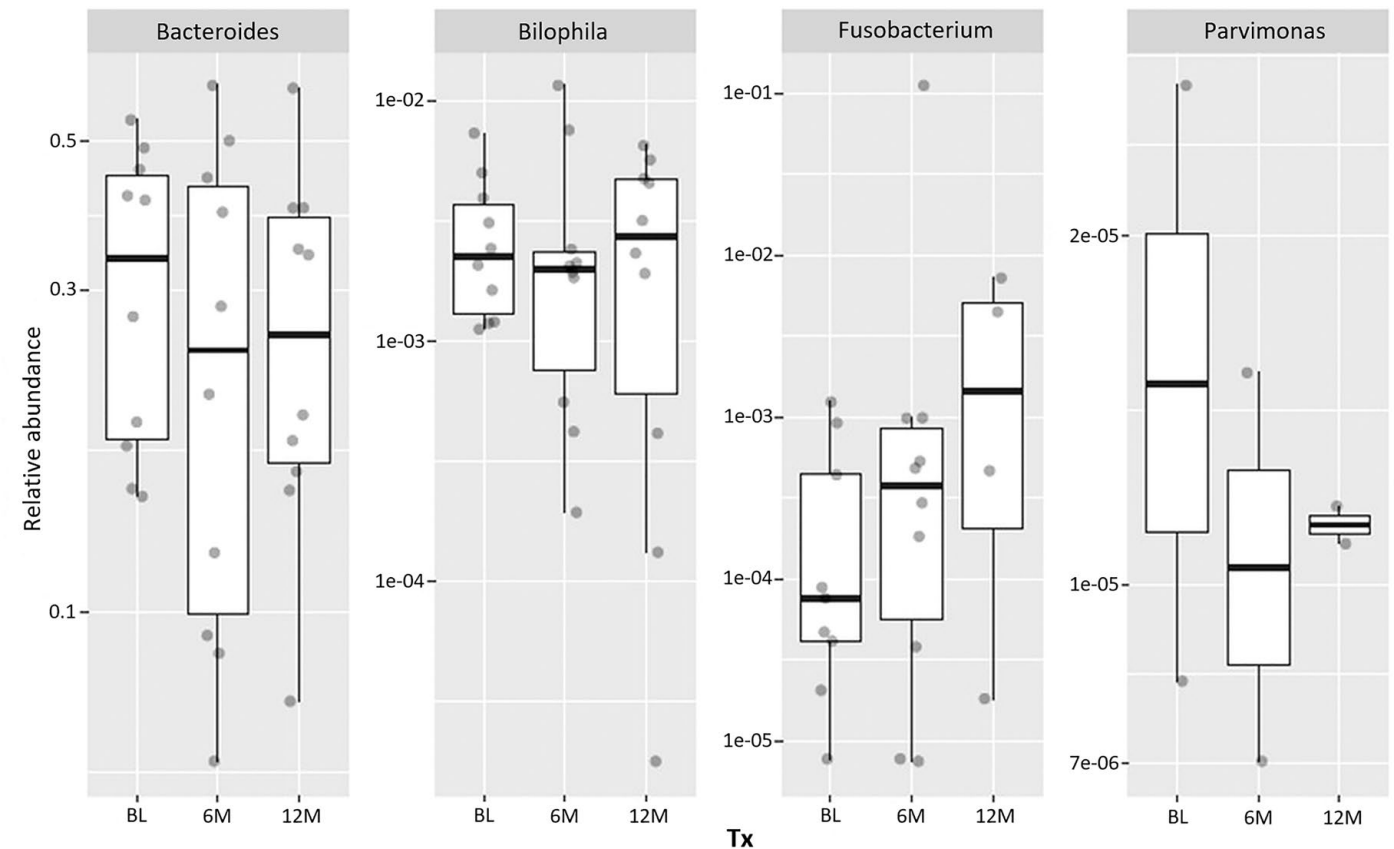
Time‐varying relative abundance of genera previously reported to be associated with colorectal cancer.

At last, we focused on the taxa that have been reported to be associated with CRC. No sequences corresponding to *Alistipes*, *Escherichia‐Shigella*, *Fusobacterium nucleatum* , *Clostridium symbiosum* , *Peptostreptococcus stomatis* , *Solobacterium moorei* , *Parvimonas micra* , or *Helicobacter pylori* were detected in any of the samples. Instead, four other genera were identified, and their relative abundance over time is displayed in Figure 5. The time‐varying changes did not show consistent patterns for most genera, except for the *Fusobacterium*, which demonstrated an increasing trend over time. However, none of these changes were statistically significant (Table S3).

## 4 Discussion

Thanks to the continuous advances in the knowledge of gut microbiota and the metabolism of bile acids, now we have a deeper understanding of their bidirectional interaction [23]. Bile acids influence the bacterial composition in gut through their antimicrobial effect and the activation of host signaling pathways. Conversely, the metabolic capacity of gut microbiota is one of the key factors that shape the bile acid composition. Previous studies indicated that antibiotics, physical activity, and diet all counted in the interplay of bile acids and gut microbiota [23, 24]. However, current evidence showed inconsistent findings regarding the impact of cholecystectomy on the change of gut microbiota. For instance, how cholecystectomy affects microbial diversity shows contradictions among three studies [4, 6, 7]. We believe this ambiguity arises partly from substantial variations in the interval between cholecystectomy and stool sampling across participants. Two additional studies, which specified the sampling times—one immediately post‐operation and the other at 3 months later—both found no significant changes in alpha diversity [5, 25]. The authors speculated that the effect of cholecystectomy on the microbiome might not be evident within 3 months. Our data, on the other hand, showed that microbial diversity decreased significantly at 6 M and returned to BL levels at 12 M. These observations suggest that post‐cholecystectomy microbiota may undergo time‐dependent fluctuations.

While most studies have reported changes in microbial composition after cholecystectomy, it remains inconclusive which species contribute most to PCD, likely due to the small sample sizes in each study. In our cohort, several genera showed temporal patterns that were broadly consistent with observations from earlier studies. For example, previous research showed that the genus *Ruminococcus* was negatively associated with defecation frequency and a looser stool form [4]. In our cohort, *Ruminococcus* was significantly lower at 6 M compared to baseline and 12 M. On the contrary, the genus *Prevotella* peaked at 6 M and was lower at BL and 12 M, a pattern linked to PCD in previous studies [4, 6]. Additionally, *Lactobacillus*, known for its role in probiotics and intestinal health, showed a bimodal change with a trough at 6 M [26]. These taxa‐level shifts may be relevant to hypotheses regarding PCD pathophysiology; however, larger and better‐controlled studies will be required to determine whether they have true clinical significance.

As the two most predominant phyla in human gut, the *Firmicutes* and *Bacteroidetes* represent over 90% of the total community [27]. A dysregulated F/B ratio is regarded as a sign of gut dysbiosis, and is associated with various pathological conditions such as obesity, hypertension, gestational diabetes, and the aging process [28, 29, 30, 31]. Patients with irritable bowel syndrome (IBS) displayed a distinct microbiota profile—increased selected Firmicutes species with depletion of Bacteroidetes in one subgroup, which could normalize under the low FODMAP diet [32]. Conversely, researches on PCD have shown a trend toward a reduced F/B ratio [4, 6]; our data also demonstrated a reduced F/B ratio at 6 M and recovery to BL levels at 12 M. This suggests that the pathophysiology of PCD and IBS might be quite different. Xu et al. had investigated the metabolic changes of fecal bile acids among PCD patients, not only revealing their distinct compositions from non‐PCD and health control groups but also presenting excessive total bile acid output in feces [4]. Moreover, their team had further shown that the signaling pathway of colonic serotonin, possibly mediated by the secondary bile acid metabolites from gut microbiota, might play an important role in inducing PCD through the mice model [33]. Still, future biological or functional analyses with shotgun metagenome sequencing are needed to better understand the mechanism of the post‐cholecystectomy gut microbiota change and its association with PCD.

The interaction between cholecystectomy, gut microbiota, and CRC is not well understood yet. One case–control study found that the gut microbiota of post‐cholecystectomy patients differed from that of healthy individuals but was similar to that of colorectal cancer (CRC) patients [15]. Previous studies showed that the abundance of *Lactobacillus* at the genus level was significantly reduced in the patients with CRC [34], and similar decreasing trend was observed in the animal model simulating the post‐cholecystectomy condition as well as in our cohort [16, 35]. Genus *Fusobacterium* was proposed to be linked to colorectal cancer through mechanisms involving the activation of the inflammatory nuclear factor‐kappa b (NF‐kB) signaling pathway and T cell‐mediated adaptive immune response [19]. An increased abundance of *Fusobacterium* has been observed in post‐cholecystectomy patients and is hypothesized to promote the development of CRC [15]. In our cohort, we also noted a rising trend in *Fusobacterium*, though it was not statistically significant. Therefore, any potential link to CRC risk remains theoretical based on our data. Given the relatively short follow‐up period of our study compared to the disease course of CRC and the absence of CRC patients in our cohort, further research is needed to confirm whether these microbial changes are linked to CRC.

There are some limitations to our study. First, the small sample size may have made the results more susceptible to individual variability and limited the generalizability to a broader population. Second, we didn't analyze gut microbiota at 1 M, when participants experienced the most significant changes in their Bristol stool scale. This hindered our ability to explore the correlation between microbial changes and changes in bowel habits, making it difficult to establish a causal relationship. Third, the absence of a non‐surgical control group makes it difficult to distinguish surgery‐induced changes from normal temporal variations or environmental factors. Fourth, we did not control for potential confounders such as dietary changes or medication use post‐surgery, which are known to impact the gut microbiota. To address these limitations, future studies with larger cohorts, additional stool analyses at multiple time points, and health control groups are needed to further validate these findings and gain a deeper understanding of the mechanisms linking gut microbiota changes to post‐cholecystectomy diarrhea.

Our study observed the post‐cholecystectomy change of microbial diversity and composition. These preliminary findings provide a basis for hypothesizing potential links between gut dysbiosis, PCD, and an increased risk of CRC, though causal relationships cannot be established at this stage. A great portion of these changes reached a peak/trough at 6 M and returned to near baseline levels at 12 M. While these observations suggest the possibility of a temporal pattern in post‐cholecystectomy microbiota dynamics, they should be interpreted cautiously given the limited sample size. Additional longitudinal studies with larger cohorts will be needed to clarify whether this pattern represents a consistent biological trend.

## Funding

This work was supported by grants from Fu Jen Catholic University and Liu‐Kung Agriculture Foundation (project number: 7100086).

## Ethics Statement

Our study was approved by the institutional ethics committee of Fu Jen Catholic University Hospital (FJUH‐IRB number: FJUH109068).

## Consent

Informed consents were obtained from all participants.

## Conflicts of Interest

The authors declare no conflicts of interest.

## Supporting information


**Table S1:** The 8 genera with significantly higher abundance at specific time points.
**Table S2:** Genera that showed marked changes over time.
**Table S3:** The time‐varying abundance of genera that had been reported to be associated with colorectal cancer.

## Data Availability

The datasets generated and/or analyzed during the current study are available in the BioProject repository under ID: PRJNA1188648.

## References

[1] X. Wang , W. Yu , G. Jiang , et al., “Global Epidemiology of Gallstones in the 21st Century: Systematic Review and Meta‐Analysis,” Clinical Gastroenterology and Hepatology 22, no. 8 (2024): 1586–1595. 38382725 10.1016/j.cgh.2024.01.051

[2] M. Fisher , D. C. Spilias , and L. K. Tong , “Diarrhoea After Laparoscopic Cholecystectomy: Incidence and Main Determinants,” ANZ Journal of Surgery 78, no. 6 (2008): 482–486. 18522570 10.1111/j.1445-2197.2008.04539.x

[3] G. Sciarretta , A. Furno , M. Mazzoni , and P. Malaguti , “Post‐Cholecystectomy Diarrhea: Evidence of Bile Acid Malabsorption Assessed by SeHCAT Test,” American Journal of Gastroenterology 87, no. 12 (1992): 1852–1854. 1449156

[4] Y. Xu , H. Jing , J. Wang , et al., “Disordered Gut Microbiota Correlates With Altered Fecal Bile Acid Metabolism and Post‐Cholecystectomy Diarrhea,” Frontiers in Microbiology 13 (2022): 800604. 35250923 10.3389/fmicb.2022.800604 PMC8894761

[5] C. K. Noh , W. Jung , M. J. Yang , W. H. Kim , and J. C. Hwang , “Alteration of the Fecal Microbiome in Patients With Cholecystectomy: Potential Relationship With Postcholecystectomy Diarrhea – Before and After Study,” International Journal of Surgery 109, no. 9 (2023): 2585–2597. 37288587 10.1097/JS9.0000000000000518 PMC10498850

[6] Y. D. Li , B. N. Liu , S. H. Zhao , Y. L. Zhou , L. Bai , and E. Q. Liu , “Changes in Gut Microbiota Composition and Diversity Associated With Post‐Cholecystectomy Diarrhea,” World Journal of Gastroenterology 27, no. 5 (2021): 391–403. 33584071 10.3748/wjg.v27.i5.391 PMC7856843

[7] W. J. Yoon , H. N. Kim , E. Park , et al., “The Impact of Cholecystectomy on the Gut Microbiota: A Case‐Control Study,” Journal of Clinical Medicine 8, no. 1 (2019): 79. 30641967 10.3390/jcm8010079 PMC6352247

[8] T. P. Yueh , F. Y. Chen , T. E. Lin , and M. T. Chuang , “Diarrhea After Laparoscopic Cholecystectomy: Associated Factors and Predictors,” Asian Journal of Surgery 37, no. 4 (2014): 171–177. 24647139 10.1016/j.asjsur.2014.01.008

[9] S. D. Hearing , L. A. Thomas , K. W. Heaton , and L. Hunt , “Effect of Cholecystectomy on Bowel Function: A Prospective, Controlled Study,” Gut 45, no. 6 (1999): 889–894. 10562588 10.1136/gut.45.6.889 PMC1727745

[10] G. A. Kullak‐Ublick , G. Paumgartner , and F. Berr , “Long‐Term Effects of Cholecystectomy on Bile Acid Metabolism,” Hepatology 21, no. 1 (1995): 41–45. 7806167 10.1002/hep.1840210109

[11] L. Vernick and L. Kuller , “Cholecystectomy and Right‐Sided Colon Cancer: An Epidemiological Study,” Lancet 318, no. 8243 (1981): 381–383. 10.1016/s0140-6736(81)90830-8 6115157

[12] J. Lagergren , W. Ye , and A. Ekbom , “Intestinal Cancer After Cholecystectomy: Is Bile Involved in Carcinogenesis?,” Gastroenterology 121, no. 3 (2001): 542–547. 11522737 10.1053/gast.2001.27083

[13] L. Yu , W. Liu , Y. Yan , Y. Jiang , X. Gao , and S. Ruan , “No Association Between Cholecystectomy and Risk of Colorectal Cancer: A Meta‐Analysis of Cohort Studies,” International Journal of Colorectal Disease 38, no. 1 (2023): 179. 37368048 10.1007/s00384-023-04463-0

[14] Y. K. Chen , J. H. Yeh , C. L. Lin , et al., “Cancer Risk in Patients With Cholelithiasis and After Cholecystectomy: A Nationwide Cohort Study,” Journal of Gastroenterology 49, no. 5 (2014): 923–931. 23807230 10.1007/s00535-013-0846-6

[15] X. Ren , J. Xu , Y. Zhang , et al., “Bacterial Alterations in Post‐Cholecystectomy Patients Are Associated With Colorectal Cancer,” Frontiers in Oncology 10 (2020): 1418. 32903396 10.3389/fonc.2020.01418 PMC7434860

[16] X. Jiang , Z. Jiang , Q. Cheng , W. Sun , M. Jiang , and Y. Sun , “Cholecystectomy Promotes the Development of Colorectal Cancer by the Alternation of Bile Acid Metabolism and the Gut Microbiota,” Frontiers in Medicine 9 (2022): 1000563. 36213655 10.3389/fmed.2022.1000563 PMC9540502

[17] T. N. Y. Kwong , X. Wang , G. Nakatsu , et al., “Association Between Bacteremia From Specific Microbes and Subsequent Diagnosis of Colorectal Cancer,” Gastroenterology 155, no. 2 (2018): 383–390. 29729257 10.1053/j.gastro.2018.04.028

[18] J. Yu , Q. Feng , S. H. Wong , et al., “Metagenomic Analysis of Faecal Microbiome as a Tool Towards Targeted Non‐Invasive Biomarkers for Colorectal Cancer,” Gut 66, no. 1 (2017): 70–78. 26408641 10.1136/gutjnl-2015-309800

[19] M. T. Weng , Y. T. Chiu , P. Y. Wei , C. W. Chiang , H. L. Fang , and S. C. Wei , “Microbiota and Gastrointestinal Cancer,” Journal of the Formosan Medical Association 118 (2019): S32–S41. 30655033 10.1016/j.jfma.2019.01.002

[20] C. Quast , E. Pruesse , P. Yilmaz , et al., “The SILVA Ribosomal RNA Gene Database Project: Improved Data Processing and Web‐Based Tools,” Nucleic Acids Research 41, no. D1 (2013): D590–D596. 23193283 10.1093/nar/gks1219 PMC3531112

[21] J. N. Paulson , O. C. Stine , H. C. Bravo , and M. Pop , “Differential Abundance Analysis for Microbial Marker‐Gene Surveys,” Nature Methods 10, no. 12 (2013): 1200–1202. 24076764 10.1038/nmeth.2658 PMC4010126

[22] S. Dray and A. B. Dufour , “The ade4 Package: Implementing the Duality Diagram for Ecologists,” Journal of Statistical Software 22, no. 4 (2007): 1–20.

[23] S. L. Collins , J. G. Stine , J. E. Bisanz , C. D. Okafor , and A. D. Patterson , “Bile Acids and the Gut Microbiota: Metabolic Interactions and Impacts on Disease,” Nature Reviews Microbiology 21, no. 4 (2023): 236–247. 36253479 10.1038/s41579-022-00805-x PMC12536349

[24] E. Molina‐Molina , R. Lunardi Baccetto , D. Q. Wang , O. de Bari , M. Krawczyk , and P. Portincasa , “Exercising the Hepatobiliary‐Gut Axis. The Impact of Physical Activity Performance,” European Journal of Clinical Investigation 48, no. 8 (2018): e12958. 29797516 10.1111/eci.12958 PMC8118139

[25] I. Grigor'eva , T. Romanova , N. Naumova , T. Alikina , A. Kuznetsov , and M. Kabilov , “Gut Microbiome in a Russian Cohort of Pre‐ and Post‐Cholecystectomy Female Patients,” Journal of Personalized Medicine 11, no. 4 (2021): 294. 33921449 10.3390/jpm11040294 PMC8070538

[26] R. Huang , F. Wu , Q. Zhou , et al., “Lactobacillus and Intestinal Diseases: Mechanisms of Action and Clinical Applications,” Microbiological Research 260 (2022): 127019. 35421680 10.1016/j.micres.2022.127019

[27] J. Qin , R. Li , J. Raes , et al., “A Human Gut Microbial Gene Catalogue Established by Metagenomic Sequencing,” Nature 464, no. 7285 (2010): 59–65. 20203603 10.1038/nature08821 PMC3779803

[28] R. E. Ley , P. J. Turnbaugh , S. Klein , and J. I. Gordon , “Microbial Ecology: Human Gut Microbes Associated With Obesity,” Nature 444, no. 7122 (2006): 1022–1023. 17183309 10.1038/4441022a

[29] T. Yang , M. M. Santisteban , V. Rodriguez , et al., “Gut Dysbiosis Is Linked to Hypertension,” Hypertension 65, no. 6 (2015): 1331–1340. 25870193 10.1161/HYPERTENSIONAHA.115.05315 PMC4433416

[30] P. Sililas , L. Huang , C. Thonusin , et al., “Association Between Gut Microbiota and Development of Gestational Diabetes Mellitus,” Microorganisms 9, no. 8 (2021): 1686. 34442765 10.3390/microorganisms9081686 PMC8400162

[31] A. Vaiserman , M. Romanenko , L. Piven , et al., “Differences in the Gut Firmicutes to Bacteroidetes Ratio Across Age Groups in Healthy Ukrainian Population,” BMC Microbiology 20, no. 1 (2020): 221. 32698765 10.1186/s12866-020-01903-7 PMC7374892

[32] K. Vervier , S. Moss , N. Kumar , et al., “Two Microbiota Subtypes Identified in Irritable Bowel Syndrome With Distinct Responses to the Low FODMAP Diet,” Gut 71, no. 9 (2022): 1821–1830. 34810234 10.1136/gutjnl-2021-325177 PMC9380505

[33] Y. Xu , J. Wang , X. Wu , et al., “Gut Microbiota Alteration After Cholecystectomy Contributes to Post‐Cholecystectomy Diarrhea via Bile Acids Stimulating Colonic Serotonin,” Gut Microbes 15, no. 1 (2023): 2168101. 36732497 10.1080/19490976.2023.2168101 PMC9897804

[34] C. Y. Fang , J. S. Chen , B. M. Hsu , B. Hussain , J. Rathod , and K. H. Lee , “Colorectal Cancer Stage‐Specific Fecal Bacterial Community Fingerprinting of the Taiwanese Population and Underpinning of Potential Taxonomic Biomarkers,” Microorganisms 9, no. 8 (2021): 1548. 34442626 10.3390/microorganisms9081548 PMC8401100

[35] H. Cao , M. Xu , W. Dong , et al., “Secondary Bile Acid‐Induced Dysbiosis Promotes Intestinal Carcinogenesis,” International Journal of Cancer 140, no. 11 (2017): 2545–2556. 28187526 10.1002/ijc.30643

