# Supplementary material for: The Post‐Cholecystectomy Gut Microbiota Follows a Time‐Varying Change—A Pilot Study
Source: JGH Open. 2025 Dec 28;9(12):e70329. doi: 10.1002/jgh3.70329 (PMC12744953; doi:10.1002/jgh3.70329)
Supplement: Supplementary file 1 — Table S1: The 8 genera with significantly higher abundance at specific time points. Table S2: Genera that showed marked changes over time. Table S3: The time‐varying abundance of genera that had been reported to be associated with colorectal cancer. [file JGH3-9-e70329-s001.docx]

**Supplementary Digital Content 1.** Bioinformatics analysis

The hypervariable regions V3–V4 of bacterial 16S rRNA genes were amplified by PCR using bar-coded universal primers 341F (F, forward primer; 5'CCTACgggNggCWgCAg-3') and 805R (R, reverse primer; 5'gACTACHCgggTATCTAATCC-3'). Library construction and sequencing of amplicon DNA samples were done by Genomics BioScience (New Taipei City, Taiwan). A paired-end library was constructed using MiSeq Reagent Kit v3 according to the manufacturer’s instructions (Illumina, Wilmington, DE, USA), and high-throughput sequencing was performed on Illumina MiSeq platform (Illumina). The bioinformatics analysis of 16S rRNA amplicon was performed by Germark Biotechnology (Taichung, Taiwan). Briefly, USEARCH (v7.0.1090) was used to merge paired-end reads on a per-sample basis, setting 8 bp as the minimum overlap of read pair. Mothur (v1.34.3) was used for merging and quality-filtering of reads to maintain reads at between 400 bp and 550 bp, with minimum average quality score ≥27. We excluded reads containing an ambiguous base or homopolymer exceeding 8 bp. Chimera detection was conducted via USEARCH (reference mode and 3% minimum divergence). Quality-filtered and non-chimeric reads were analyzed (UPARSE pipeline) to generate operational taxonomic units (OTUs) per sample (97% identity level). OTU representative were searched against the Greengenes 13_5 database using USEARCH global alignment to identify the corresponding taxonomy of the best hit. Any OTU without a hit or with only a weak hit (i.e., the average of % sequence identity and % alignment coverage < 93) was excluded from the following analyses.

**Supplementary Table 1.** The 8 genera with significantly higher abundance at specific time points.

| Bacterial taxa | LogMaxMean | Group | LDA | p-value |
| --- | --- | --- | --- | --- |
| Bacteria.Firmicutes.Clostridia.Clostridiales.Lachnospiraceae.Ruminococcus | 3.672606 | 12M | 3.28 | 0.005 |
| Bacteria.Proteobacteria.Betaproteobacteria.Burkholderiales.Burkholderiaceae.Burkholderia | 0.602716 | 12M | 2.10 | 0.040 |
| Bacteria.Firmicutes.Clostridia.Clostridiales.Lachnospiraceae.Epulopiscium | 1.903446 | 6M | 2.02 | 0.012 |
| Bacteria.Firmicutes.Bacilli.Lactobacillales.Lactobacillaceae.Lactobacillus | 3.470678 | BL | 3.15 | <0.001 |
| Bacteria.Firmicutes.Clostridia.Clostridiales.Veillonellaceae.Mitsuokella | 3.421588 | BL | 3.09 | 0.004 |
| Bacteria.Actinobacteria.Actinobacteria.Bifidobacteriales.Bifidobacteriaceae.Gardnerella | 2.81145 | BL | 2.48 | <0.001 |
| Bacteria.Firmicutes.Erysipelotrichi.Erysipelotrichales.Erysipelotrichaceae.Bulleidia | 0.358956 | BL | 2.20 | 0.040 |
| Bacteria.Proteobacteria.Betaproteobacteria.Neisseriales.Neisseriaceae.Neisseria | 2.348205 | BL | 2.12 | 0.002 |

**Supplementary Table 2.** Genera that showed marked changes over time.

|  | Genus | Meandiff | Maxnonzeroprop | p-values | adjPvalues |
| --- | --- | --- | --- | --- | --- |
| 1 | Lactobacillus | 0.00277 | 1 | <0.001 | <0.001 |
| 2 | Mituokella | 0.00264 | 0.7 | 0.003 | 0.026 |
| 3 | Prevotella^†^ | 0.14096 | 1 | 0.280 | 0.884 |
| 4 | Bacteroides | 0.04514 | 1 | 0.217 | 0.728 |
| 5 | Roseburia | 0.03043 | 1 | 0.578 | >0.999 |
| 6 | [Prevotella]^†^ | 0.02242 | 1 | 0.136 | 0.509 |
| 7 | Phascolarctobacterium | 0.01632 | 1 | 0.820 | >0.999 |
| 8 | Sutterella | 0.01484 | 1 | 0.854 | >0.999 |
| 9 | Parabacteroides | 0.01411 | 1 | 0.250 | 0.814 |
| 10 | Fusobacterium | 0.01126 | 1 | 0.386 | >0.999 |

†. Prevotella:
Bacteria.Bacteroiddetes.Bacteroidia.Bacteroidales.Prevotellaceae.Prevotella;

† [Prevotella]: Bacteria.Bacteroiddetes.Bacteroidia.Bacteroidales.Paraprevotellaceae.Prevotella

**Supplementary Table 3.** The time-varying abundance of genera that had been reported to be associated with colorectal cancer

| Rank | Tax | Median test p-value | Mean  test p-value | Trend  lm.beta^†^ | Trend p-value |
| --- | --- | --- | --- | --- | --- |
| Genus | Bacteroides | 0.68 | 0.81 | -0.02 | 0.59 |
| Genus | Bilophila | 0.90 | 0.99 | <0.01 | 0.97 |
| Genus | Fusobacterium | 0.12 | 0.40 | <0.01 | 0.92 |
| Genus | Parvimonas | >0.99 | 0.88 | >-0.01 | 0.66 |

†. This field represents the standardized regression coefficient, obtained by applying the lm.beta function from the R package lm.beta to linear model objects (in this case, the relative abundance of each genus).
